# Association between taste receptor (TAS) genes and the perception of wine characteristics

**DOI:** 10.1038/s41598-017-08946-3

**Published:** 2017-08-23

**Authors:** Maura Carrai, Daniele Campa, Pavel Vodicka, Riccardo Flamini, Irene Martelli, Jana Slyskova, Katerina Jiraskova, Alexandra Rejhova, Sona Vodenkova, Federico Canzian, Alberto Bertelli, Antonio Dalla Vedova, Luigi Bavaresco, Ludmila Vodickova, Roberto Barale

**Affiliations:** 1 Department of Biology, Pisa University, Pisa, Italy; 2 Department of Molecular Biology of Cancer, Institute of Experimental Medicine, Academy of Science of Czech Republic, Prague, Czech Republic; 3 Institute of Biology and Medical Genetics, 1st Medical Faculty, Charles University, Prague, Czech Republic; 4 Biomedical Centre, Medical School Pilsen, Charles University in Prague, Pilsen, Czech Republic; 5 CREA - Research Centre for Viticulture, Conegliano, Italy; 6 Sistemi Territoriali S.r.l., 56021 Cascina Loc, San Prospero Italy; 7 1st Medical Faculty, Charles University in Prague, 12000 Prague, Czech Republic; 8 Genomic Epidemiology Group, German Cancer Research Center, Heidelberg, Germany; 9 Department of Biomedical Sciences for Health, Università degli Studi di Milano, Milan, Italy; 10 Department of Sustainable Crop Production, Pomology and Viticulture Section, Università Cattolica del Sacro Cuore, Piacenza, Italy

## Abstract

Several studies have suggested a possible relationship between polymorphic variants of the taste receptors genes and the acceptance, liking and intake of food and beverages. In the last decade investigators have attempted to link the individual ability to taste 6-n-propylthiouracil (PROP) and the sensations, such as astringency and bitterness, elicited by wine or its components, but with contradictory results. We have used the genotype instead of the phenotype (responsiveness to PROP or other tastants), to test the possible relation between genetic variability and the perception of wine characteristic in 528 subjects from Italy and the Czech Republic. We observed several interesting associations, among which the association between several *TAS2R38* gene single nucleotide polymorphisms (P = 0.002) and the *TAS2R16-*rs6466849 polymorphism with wine sourness P = 0.0003). These associations were consistent in both populations, even though the country of origin was an important factor in the two models, thus indicating therefore that genetics alongside cultural factors also play a significant role in the individual liking of wine.

## Introduction

Red wine represents a common component of the Mediterranean diet which is associated with reduced mortality from cardiovascular disease as well as increased longevity^1, 2^. Part of the beneficial effects of moderate red wine drinking is thought to be due to the presence of bioactive compounds, mainly polyphenols, representing a source of beneficial antioxidant compounds^3^. Some of them, catechins, epicatechin, tannins, hydrocinnamic acids, and other phenolic compounds, also confer to wine its taste^1, 4, 5^.

In the last twenty years several studies have attempted to link individual ability to taste 6-n-propylthiouracil (PROP) and the sensations, such as astringency and bitterness, generated by wine or its components, but with inconsistent results^6–9^. Bitterness in red wine is mainly induced by polyphenols as recently reviewed by Soares and colleagues^10^. The authors describe several molecules such as tannins, polymeric fractions of tannic acid, flavan-3-ols, anthocyanins, cathechin, procyanidins and also point out that genetic variability may play a role in red wine bitterness perception. Astringency in red wine is less known than bitterness but it is believed to be manly the result of tannins interaction with an array of proteins present in the saliva. There are at least 25 functional *TAS2R* genes in humans, whose products are responsible for bitter perception^11–14^. These genes are highly polymorphic, probably to provide a wide range of phenotypic response to bitter stimuli^15, 16^. *TAS2R* genes have been profoundly studied in relation to the susceptibility of several chronic diseases and other human traits^17–27^. Several studies have suggested a possible relationship between polymorphic variants in taste genes and food and beverage acceptance, liking and intake^6, 28–35^. The genetic variability of taste receptors has also been suggested to affect alcohol intake. For example, lower alcohol consumption was observed in individuals carrying the PAV haplotype of the *TAS2R38* gene, whereas individuals with the ancestral T allele of rs846664-*TAS2R16* gene showed an increased risk of alcohol dependence^36–38^. In addition Lanier and colleagues reported that sweet and bitter tastes of alcoholic beverages may mediate alcohol intake^38^. Genetic variability has also been suggested to be associated with alcohol abuse^39, 40^.

To further our understanding on wine acceptance or preference we have used, for the first time, a Mendelian randomization approach. We tested the possible relation between 29 taste receptor (TAS) single nucleotide polymorphisms (SNP) and the perception of wine characteristics. This approach is also supported by a recent report in which the authors identified several TAS genes, such as *TAS2R4*, *TAS2R5*, *TAS2R39* and *TAS2R7*, to be activated by polyphenol compounds that are frequently present in red wine^41^.

## Results

### Data filtering and quality controls

All SNPs were in Hardy Weinberg equilibrium (HWE) with the exception of *TAS2R7* - rs2588350) and *TAS2R1* - rs2234233 that were therefore excluded from further analyses. *TAS1R2* - rs4920566 and CA6 - rs2274333 were not genotyped successfully in the Italian population, therefore all the results regarding these two SNPs refer to the Czech population only. All samples that had a genotyping call rate lower than 75% (N = 71) were not further analysed. For the remaining samples (N = 528), the median genotyping call was 96.77%. The final number of participants who were eligible for association studies between genotype and phenotype (fully characterized by the questionnaire, stimuli perception and genotyping), included 272 Czechs (average age 43.4 ± 11.7; male 58%, female 42%) and 235 Italians (average age 37.7 ± 15.2; male 47%, female 53%). The association analyses were carried out using 27 SNPs belonging to 20 genes in a total number of 507 individuals. The study-wide Meff was 25.196, resulting in a threshold for significance, after the correction for multiple comparisons, of p = 0.05/25.196 = 0.002.

### Associations between PROP sensitivity and wine descriptors

We observed a statistically significant inverse correlation between PROP non-taster status and the level of bitterness perceived in the wine. This association was consistent in the Italian population, in the Czech population and in the pooled analysis, but it was statistically significant at the 0.05 level only in the Italian and in pooled groups, with coefficients −0.26 (95% CI −0.46; −0.06, P = 0.01) and −0.20 (95% CI −0.34; −0.06, P = 0.005) respectively. The Czech population showed the same trend but did not reach statistical significance: coefficient −0.17 (95% CI −0.37; 0.03), P = 0.09. There was no association between PROP tasting phenotype and the perceived astringency and sourness of the wine in any of the strata analysed. The results are shown in Table 1.

**Table 1:** Associations between PROP sensitivity and wine descriptors.

Wine descriptors	Czech Republic			Italy			Czech Republic and Italy		
	Coef.	95% CI	P-value	Coef.	95% CI	P-value	Coef.	95% CI	P-value
Bitterness	−0.17	(−0.37; 0.03)	0.09	−0.26	(−0.46; −0.06)	0.01	−0.20	(−0.34; −0.06)	0.005
Sourness	0.13	(−0.07; 0.32)	0.20	−0.11	(−0.29; 0.07)	0.23	0.01	(−0.12; 0.14)	0.85
Astringency	0.08	(−0.09; 0.25)	0.34	−0.17	(−0.35; 0.01)	0.07	−0.02	(−0.15; 0.10)	0.71

### Associations between PROP sensitivity and *TAS2R38* SNPs

We tested, as a proof of principle, the associations between the selected SNPs and PROP phenotype. As expected, we observed strong statistical associations for *TAS2R38-*rs10246939, *TAS2R38-*rs172686 and *TAS2R38-*rs713598, as shown in Table 2. We also observed an association between *TAS2R5*-rs2227264 and PROP phenotype, however this SNP and the variants in the *TAS2R38* gene are in linkage disequilibrium (D’ = 0.67 In 1000 G for Caucasian) and reflect the same signal Supplementary Table S1.

**Table 2:** Associations between *TAS3R38* SNPs and PROP phenotype.

Gene	SNP	Model	Czech Republic			Italy			Czech Republic and Italy		
			Coef.	95% CI	P-value	Coef.	95% CI	P-value	Coef.	95% CI	P-value
*TAS2R38*	rs10246939	C/T vs C/C	0.37	(−0.16; 0.90)	0.17	0.32	(−0.19; 0.85)	0.22	0.37	(0.00; 0.74)	**0.05**
		T/T vs C/C	1.39	(0.80; 1.98)	**3.5 × 10** ^**−6**^	2.11	(1.53; 2.69)	**8.8 × 10** ^**−13**^	1.71	(1.30; 2.12)	**4.4 × 10** ^**−16**^
	rs1726866	T/C vs C/C	0.38	(−0.15; 0.90)	0.16	0.32	(−0.20; 0.85)	0.22	0.36	(0.00; 0.74)	**0.05**
		T/C vs C/C	1.42	(0.84; 2.01)	**1.9 × 10** ^**−6**^	2.10	(1.52; 2.68)	**1.5 × 10** ^**−12**^	1.72	(1.31; 2.13)	**4.4 × 10** ^**−16**^
	rs713598	G/C vs C/C	0.27	(−0.30; 0.84)	0.35	0.39	(−0.17; 0.94)	0.17	0.35	(−0.04; 0.76)	0.08
		G/G vs C/C	1.27	(0.66; 1.87)	**3.0 × 10** ^**−5**^	2.04	(1.45; 2.63)	**1.1 × 10** ^**−11**^	1.63	(1.21; 2.06)	**3.7 × 10** ^**−14**^

### Associations between SNPs and wine descriptors

We observed several promising associations between the selected polymorphic variants and the wine descriptors. The most consistent was the one between the *TAS2R38* SNPs and wine bitterness. All the variant alleles were inversely associated with bitterness perception. We recorded the strongest effect when comparing the variant homozygous T/T with the common C/C of the rs1726866 SNP: coefficient −1.16 (95% CI −2.13; −0.18), P = 0.02 (Czechs), coefficient −0.94 (95% CI −1.94; −0.05), P = 0.06 (Italians) and coefficient −1.10 (95% CI −1.80; −0.40), P = 0.002 (pooled data). The results are shown in Table 3 and in Supplementary Table S2. In addition, the variant allele of the *TAS2R16*-rs6466849 was associated with wine sourness with the strongest association observed in the pooled data: coefficient 0.94 (95% CI 0.43; 1.46), P = 0.0003. This association was consistent across all three strata but did not reach statistical significance among the Italians (Table 3 and in Supplementary Table S3). For *TAS1R2*-rs4920566 we observed a statistically significant association with wine astringency: coefficient −1.24 (95% CI −1.97; −0.50), P = 0.001. However this SNP was successfully genotyped only in the Czech population (Table 3 and in Supplementary Table S4). Additionally, using an unconditional logistic regression, adjusting for age, gender, nationality, we observed that individuals with at least one A allele of the *TAS2R16 −* rs6466849 had a decreased, although not significant, tendency of drinking wine OR 0.65 (95% CI; 0.40–1.04, P = 0.071). By stratifying for gender this association was statistically significant only in females subjects (p = 0.005) suggesting a possible interaction between gender and wine drinking.

**Table 3:** Statistically significant associations between *TAS* genes polymorphisms and wine descriptors.

*Bitterness*				Czech Republic			Italy			Czech Republic and Italy		
Chr.7	Gene	SNP	Model	Coef.	95% CI	P-value	Coef.	95% CI	P-value	Coef.	95% CI	P-value
	*TAS2R38*	rs10246939	C/T vs C/C	−0.90	(−1.78; −0.01)	0.05	−0.06	(−0.96; 0.84)	0.90	−0.54	(−1.17; 0.09)	0.10
			T/T vs C/C	−1.12	(−2.11; −0.14)	0.03	−0.97	(−1.96; 0.02)	0.06	−1.08	(−1.78; −0.38)	**0.003**
		rs1726866	T/C vs C/C	−1.06	(−1.94; −0.19)	0.02	−0.07	(−0.97; 0.83)	0.89	−0.63	(−1.27; −0.001)	0.05
			T/C vs C/C	−1.16	(−2.13; −0.18)	0.02	−0.94	(−1.94; 0.05)	0.06	−1.10	(−1.80; −0.40)	**0.002**
		rs713598	G/C vs C/C	−0.71	(−1.67; −0.26)	0.15	0.08	(−0.87; 1.03)	0.87	−0.34	(−1.02; 0.34)	0.33
			G/G vs C/C	−0.91	(−1.93–0.10)	0.08	−0.51	(−1.51; 0.50)	0.32	−0.75	(−1.46; −0.03)	0.04
***Sourness***				**Czech Republic**			**Italy**			**Czech Republic and Italy**		
**Chr.7**	***Gene***	**SNP**	**Model**	**Coef**.	**95% CI**	**P-value**	**Coef**.	**95% CI**	**P-value**	**Coef**.	**95% CI**	**P-value**
	*TAS2R16*	rs6466849	A/G vs G/G	1.20	(0.50; 1.91)	**0.001**	0.61	(−0.14; 1.35)	0.11	0.94	(0.43; 1.46)	**0.0003**
			A/A vs G/G	−0.75	(−2.56; 1.05)	0.41	−5.11	(−9.93; 0.28)	0.04	−1.30	(−2.94; 0.34)	0.12
***Astringency***				**Czech Republic**			**Italy**			**Czech Republic and Italy**		
**Chr.1**	***Gene***	**SNP**	**Model**	**Coef**.	**95% CI**	**P-value**	**Coef**.	**95% CI**	**P-value**	**Coef**.	**95% CI**	**P-value**
	*TAS1R2*	rs4920566	G/A vs G/G	0.29	(−0.38; 0.96)	0.40	n.a.	n.a.	n.a.	n.a.	n.a.	n.a.
			A/A vs G/G	−1.24	(−1.97; −0.50)	**0.001**	n.a.	n.a.	n.a.	n.a.	n.a.	n.a.

### Possible functional effects

We used several bioinformatic tools to predict possible functional relevance for *TAS2R16*-rs6466849. RegulomeDB showed a score of 6 suggesting minimal binding evidence. HaploReg suggested that this polymorphism alters the sequence recognized by the transcription factors SMC3_disc2, AIRE_1 and Myb_1. No significant association between rs6466849 and expression of any gene in any of the available tissues was reported in the GTEx project.

## Discussion

In this study we found a statistically significant association between PROP and wine bitterness, confirming previous observations by Pickering and colleagues^8^. We did not, however, identify any association between PROP sensitivity and wine astringency and sourness as reported in that same study^8^. This divergence in the results may be explained by the different methods used to rank the wine descriptors or by the different number of volunteers involved in the two studies with ours being, by far, the larger of the two. The novelty of the present study is the use of a Mendelian randomization approach to directly assess the effect of genetic variability on the perception of wine tastes. We observed two potentially relevant signals, namely the associations between the *TAS2R38* SNPs and *TAS2R16*-rs6466849 with wine bitterness and sourness respectively. The association between *TAS2R38* polymorphisms and wine bitterness is consistent in the three strata analyzed (Czechs alone, Italians alone and the two population together) indicating that *TAS2R38* “non tasters” have a tendency to judge the wine less bitter. Among the Italians, this association did not reach statistical significance suggesting a stronger effect of cultural and lifestyle habits in that country, which certainly play a relevant role in wine perception. Our findings are consistent with two studies^42, 43^ that reported that the bitterness of ethanol solutions differed by *TAS2R38* polymorphisms. There are overwhelming evidences supporting a strong functional effect of the *TAS2R38* SNPs we studied, which have been correlated with multiple human traits. Our report is, however the first one to highlight the possible involvement of the polymorphic variants in wine sensing. The other novel finding of this study is represented by the association between the *TAS2R16*-rs6466849 SNP and wine sourness. The above gene is known to encode a receptor able to bind to a variety of natural and synthetic bitter compounds^14^. Its polymorphic variants have been associated with a variety of human traits and phenotypes, such as nicotine dependence, ageing and alcohol consumption^7, 20, 37^. *TAS2R16*-rs6466849 is situated approximately 1000 base pairs from the 3’ end of the gene and bioinformatic tools used to assess its potential functional relevance did not help in elucidating its role in gene function. Considering also that wine is a complex blend of various molecules it is difficult to understand the exact mechanism underlying the association. However, given the strong statistical effect, it might be worth performing further studies to identify which molecules the receptor binds to, and how the polymorphic variant modifies the binding. Given that in a report by Hayes and colleagues^44^ the genetic variability of the *TAS2R16* gene was associated with alcohol intake, we tested, as an explanatory analysis, the possible relations between wine consumption, sourness and *TAS2R16* genotypes. We observed that carriers of the G allele of the *TAS2R16*-rs6466849 showed a borderline tendency of drinking less wine. The polymorphic variant analyzed by Hayes and colleagues (rs846672) is in Linkage Disequilibrium (LD) with rs6466849 therefore the findings of the two studies likely represent the same signal and support each other. Assuming that sourness in red wine is generally perceived as a negative sensation, the different sourness experienced by carriers of the different genotypes of rs6466849 might influence alcohol liking and subsequently drinking behavior. However since we could not observe a direct association between sourness sensing and alcohol intake we lack statistical evidence to support the association between rs6466849 and alcohol liking. This hypothesis, therefore, remains highly speculative. A limitation of the study is that we performed several tests and this mitigates the robustness of the outcome. In summary using a Mendelian randomization approach we have shown a suggestive direct relation between genetic variability of taste receptors genes and wine perception. In this study we have used two populations from Europe that are similar in allelic frequencies for taste receptors, but have different dietary habits (Mediterranean *Vs*. central European) and we observed that one locus (*TAS2R38*-SNP variants) was associated with wine bitterness and another one (*TAS2R16*-rs6466849) with wine sourness. These associations were consistent in both populations, even if the country of origin was an important factor in the two models, thus indicating that genetics alongside cultural factors also play a significant role in individual liking of wine.

## Material and Methods

### Study population

A sample of 599 individuals of legal drinking age composed by students, university staff and blood donors, was recruited in two different European countries: 299 at the Institute of Experimental Medicine, Czech Academy of Sciences, Prague, Czech Republic and 300 at the Biology Department, Pisa University, Italy. Volunteers were enrolled to obtain two groups with similar mean age (40 ± 15 years), and an equal male: female ratio. Participants were asked to complete a brief questionnaire including height, weight, age, gender, smoking (number of cigarette/day, cigar, pipe), drinking (wine, beer, spirits, and glass/day or by week) and ethnicity (i.e. origin of grandparents). All participants signed a written informed consent. The study was approved by ethical review boards of the institutions responsible for subject recruitment in each of the recruitment center (University of Pisa and the Czech Academy of Sciences). All procedures performed were in accordance with the 1964 Helsinki declaration and its later amendments or comparable ethical standards.

### Determination of the PROP phenotype

The individual taste determination of the PROP phenotype was determined using the staircase method. Perception response was determined using filter paper disks (2.3 cm diameter) impregnated with 100 µl solutions containing seven increasing concentrations of PROP (0.05 mM, 0.1 mM, 0.25 mM, 0.5 mM, 3 mM, 5 mM, 10 mM). The disks were dried at room temperature in the dark. Paper disks were stored in sealed plastic bags, in the dark, at room temperature up to two weeks before use; all paper disks were prepared in Pisa and half of them were immediately sent to Prague in order to avoid any bias in the preparation process. Volunteers were asked not to smoke, eat and drink coffee at least two hours before the sessions that were held in a dedicated silent room, that was adequately wide in order for volunteers not to disturb each other. Each volunteer had his/her own desk, and was assisted by qualified personnel throughout the session. After a brief description of the taste anatomy and physiology, volunteers (about 10–15 per session) were invited to place the paper disk on their tongues, to moisten it with saliva and move the paper in different parts of the mouth using the tongue mimicking chewing for at least 10 seconds. Firstly, they started with an empty paper disk in order to be trained to assess the “empty paper” taste. Then they were invited to use the same procedure to taste paper disk with increasing concentration (as described above) and to stop as soon as they had an unambiguous bitter perception of a taste. In case of substantial uncertainty (no perception or questionably detectable), volunteers were invited to taste the next concentration, and if perceived, it represented the threshold level.

### Wine selection and scaling methodology

A panel of experts from the CREA-Research Centre for Viticulture at Conegliano, chose Piave Raboso DOC red wine, 2008 vintage, produced from the native grape variety Raboso Piave (*V. vinifera* L.). It is cultivated in the Piave Denomination of Controlled Origin area (Veneto region, Italy), for its defined taste personality and mildness in aroma, suitable to decrease the confounding effects of olfaction. These characteristics make it easier for non-experts to evaluate the three chosen indicators: astringency, bitterness and sourness of wine. After an explanation of the three tastes to be assessed in the wine, the volunteers were provided with some examples: strong coffee and dark chocolate without sugar for bitterness; vinegar for sourness and raw artichoke and immature apple for astringency^45^. The participants were then invited to taste the red wine keeping it in the mouth for at least 7–10 seconds and moving it forward and backward. Next, they were asked to indicate the degree of bitterness, sourness and astringency using a visual analogic scale (VAS) from 0 (not detectable) to 10 (strong taste impression).

### Selection of tagging SNPs

Our aim was to consider genes likely to be involved in the human bitter (*TAS2Rs*) and sweet (*TAS1Rs*) taste receptor system, including functional polymorphisms belonging to genes involved in transduction pathway such as guanine nucleotide binding protein, alpha transducing 3 (*GNAT3*) and carbonic anhydrase VI (*CA6*). Therefore, according to the algorithm described by Carlson and coworkers^46^, we followed a hybrid tagging-functional method, conceived to select the maximum informative set of tag SNPs in a candidate gene/candidate region for an association study. All the considered polymorphisms exhibited a minor allele frequency (MAF) ≥ 5% in Caucasians, as reported by the International HapMap Project (version 28, August 2010; http://www.hapmap.org). Tagging SNPs were selected with the use of the Tagger program within Haploview (http://www.broad.mit.edu/mpg/haploview/; http://www.broad.mit.edu/mpg/tagger/)^47, 48^ using pairwise tagging with a minimum r^2^ of 0.8. The resulting SNPs captured genetic variability in the four regions of interest. On chromosome 5, we considered a 1440 base-pair region spanning from rs41467 to rs2234233 in the *TAS2R1* gene. For genes situated on chromosome 1, 7 and 12, we selected tagging polymorphisms. For the *TAS2R38* gene we focused on rs713598, rs1726866 and rs10246939 as tagging SNPs given that they are all non-synonymous and functional. The final selection included 29 SNPs belonging to 22 genes. Supplementary Table S5 shows the genes and SNPs included in the study, their position in the genome, in the gene, the amino acidic change specified and the gene function.

### DNA extraction and genotyping

DNA was extracted from buccal swab following Invisorb® Spin Swab Kit protocol and from blood samples with standard proteinase K digestion followed by phenol/chloroform extraction and ethanol precipitation. Biological material was collected during one research visit. The Invisorb® Spin Swab Kits simple procedure comprises the following steps: lysis of cells with standard proteinase K, binding the genomic DNA to the membrane of a Spin Filter, washing the membrane with elimination of ethanol and finally elution of genomic DNA. DNA samples were transferred on 384 well PCR plates with duplicates (12%) as randomized quality controls on the plates, in order to guarantee and monitor the effective success of the PCR. All genotyping was carried out using KASPar (Kbioscience, Heddesdon, UK) assay, the PCR plates were read on a ViiA Real-Time PCR system using the 7 RUO Software(Applied Biosystems).

### Statistical analysis

Hardy Weinberg equilibrium (HWE) was tested in the two populations separately using a Pearson chi-square test. The associations between PROP phenotype, genetic polymorphisms and wine descriptors were calculated using a general linear model computing coefficients and 95% confidence intervals (95% CI), chi-square, ordinal, logistic and multinomial regression. Genetic data were coded using a codominant inheritance model with the most common genotype as the reference category. We performed the analysis considering the two centers separately and pooled them together. All the analyses were adjusted for age and gender. In the pooled analysis we also adjusted for country of origin. We additionally performed an unconditional logistic regression to assess the possible effect of *TAS2R16* genotypes on alcohol intake. Considering the large number of tests performed in this study, we calculated the number of effective independent variables (Meff) for each gene^49^. We obtained a study-wide Meff = 25.196, by adding up each individual gene Meff.

### Bioinformatic analysis

We used several bioinformatic tools to assess the possible functional relevance for the SNP *TAS2R16*-rs6466849, showing the most significant associations. RegulomeDB (http://regulome.stanford.edu/)^50^ and HaploReg v2B^51^ were used to identify the regulatory potential of the region nearby each SNP. The GTEx portal web site^52^ was used to identify potential associations between the SNP and expression levels of nearby genes (eQTL) in all the available tissues.

## Electronic supplementary material

Supplementary tables

## References

[1] Giacosa A (2014). Mediterranean Way of Drinking and Longevity. Critical reviews in food science and nutrition.

[2] Bertelli AA (2007). Wine, research and cardiovascular disease: instructions for use. Atherosclerosis.

[3] Corder R (2006). Oenology: red wine procyanidins and vascular health. Nature.

[4] Giacosa A (2012). Alcohol and wine in relation to cancer and other diseases. European journal of cancer prevention: the official journal of the European Cancer Prevention Organisation.

[5] Giacosa A (2013). Cancer prevention in Europe: the Mediterranean diet as a protective choice. European journal of cancer prevention: the official journal of the European Cancer Prevention Organisation.

[6] Hayes JE, Pickering GJ (2012). Wine Expertise Predicts Taste Phenotype..

[7] Ishikawa T, Noble AC (1995). Temporal Perception of Astringency and Sweetness in Red Wine. Food quality and preference.

[8] Pickering, G., Simunkovar, K. & DiBattista, D. In *Food quality and preference* Vol. 15 147–154 (2004).

[9] Smith AK, June H, Noble AC (1996). Effects of viscosity on the bitterness and astringency of grape seed tannin. Food quality and preference.

[10] Soares S, Brandao E, Mateus N, de Freitas V (2017). Sensorial properties of red wine polyphenols: Astringency and bitterness. Critical reviews in food science and nutrition.

[11] Bufe B, Hofmann T, Krautwurst D, Raguse JD, Meyerhof W (2002). The human TAS2R16 receptor mediates bitter taste in response to beta-glucopyranosides. Nature genetics.

[12] Chandrashekar J, Hoon MA, Ryba NJ, Zuker CS (2006). The receptors and cells for mammalian taste. Nature.

[13] Kim UK, Drayna D (2005). Genetics of individual differences in bitter taste perception: lessons from the PTC gene. Clinical genetics.

[14] Meyerhof W (2010). The molecular receptive ranges of human TAS2R bitter taste receptors. Chemical senses.

[15] Conte C, Ebeling M, Marcuz A, Nef P, Andres-Barquin PJ (2003). Evolutionary relationships of the Tas2r receptor gene families in mouse and human. Physiological genomics.

[16] Soranzo N (2005). Positive selection on a high-sensitivity allele of the human bitter-taste receptor TAS2R16. Current biology: CB.

[17] Adappa ND (2013). Genetics of the taste receptor T2R38 correlates with chronic rhinosinusitis necessitating surgical intervention. International forum of allergy & rhinology.

[18] Adappa ND (2014). The bitter taste receptor T2R38 is an independent risk factor for chronic rhinosinusitis requiring sinus surgery. International forum of allergy & rhinology.

[19] Akao H (2012). KIF6, LPA, TAS2R50, and VAMP8 genetic variation, low density lipoprotein cholesterol lowering response to pravastatin, and heart disease risk reduction in the elderly. Atherosclerosis.

[20] Campa D (2012). Bitter taste receptor polymorphisms and human aging. PloS one.

[21] Campa D (2010). A gene-wide investigation on polymorphisms in the taste receptor 2R14 (TAS2R14) and susceptibility to colorectal cancer. BMC medical genetics.

[22] Carrai M (2011). Association between TAS2R38 gene polymorphisms and colorectal cancer risk: a case-control study in two independent populations of Caucasian origin. PloS one.

[23] Inoue H (2013). A case study on the association of variation of bitter-taste receptor gene TAS2R38 with the height, weight and energy intake in Japanese female college students. Journal of nutritional science and vitaminology.

[24] Kulkarni GV (2013). Association of GLUT2 and TAS1R2 genotypes with risk for dental caries. Caries research.

[25] Lee RJ (2012). T2R38 taste receptor polymorphisms underlie susceptibility to upper respiratory infection. The Journal of clinical investigation.

[26] Mfuna Endam L (2014). Genetic variations in taste receptors are associated with chronic rhinosinusitis: a replication study. International forum of allergy & rhinology.

[27] Schembre SM, Cheng I, Wilkens LR, Albright CL (2013). & Marchand le, L. Variations in bitter-taste receptor genes, dietary intake, and colorectal adenoma risk. Nutrition and cancer.

[28] Colares-Bento FC (2012). Implication of the G145C polymorphism (rs713598) of the TAS2r38 gene on food consumption by Brazilian older women. Archives of gerontology and geriatrics.

[29] Duffy VB (2010). Vegetable Intake in College-Aged Adults Is Explained by Oral Sensory Phenotypes and TAS2R38 Genotype. Chemosensory perception.

[30] El-Sohemy A (2007). Nutrigenomics of taste - impact on food preferences and food production. Forum of nutrition.

[31] Gorovic N (2011). Genetic variation in the hTAS2R38 taste receptor and brassica vegetable intake. Scandinavian journal of clinical and laboratory investigation.

[32] Hayes JE, Feeney EL, Allen AL (2013). Do polymorphisms in chemosensory genes matter for human ingestive behavior?. Food quality and preference.

[33] Hayes JE, Feeney EL, Nolden AA, McGeary JE (2015). Quinine Bitterness and Grapefruit Liking Associate with Allelic Variants in TAS2R31. Chemical senses.

[34] Shen Y, Kennedy OB, Methven L (2016). Exploring the effects of genotypical and phenotypical variations in bitter taste sensitivity on perception, liking and intake of brassica vegetables in the UK. Food quality and preference.

[35] Laaksonen O, Ahola J, Sandell M (2013). Explaining and predicting individually experienced liking of berry fractions by the hTAS2R38 taste receptor genotype. Appetite.

[36] Duffy VB (2004). Bitter receptor gene (TAS2R38), 6-n-propylthiouracil (PROP) bitterness and alcohol intake. Alcoholism, clinical and experimental research.

[37] Hinrichs AL (2006). Functional variant in a bitter-taste receptor (hTAS2R16) influences risk of alcohol dependence. American journal of human genetics.

[38] Lanier SA, Hayes JE, Duffy VB (2005). Sweet and bitter tastes of alcoholic beverages mediate alcohol intake in of-age undergraduates. Physiology & behavior.

[39] Dotson CD, Wallace MR, Bartoshuk LM, Logan HL (2012). Variation in the gene TAS2R13 is associated with differences in alcohol consumption in patients with head and neck cancer. Chemical senses.

[40] Wang JC (2007). Functional variants in TAS2R38 and TAS2R16 influence alcohol consumption in high-risk families of African-American origin. Alcoholism, clinical and experimental research.

[41] Soares S (2013). Different phenolic compounds activate distinct human bitter taste receptors. Journal of agricultural and food chemistry.

[42] Allen AL, McGeary JE, Hayes JE (2014). Polymorphisms in TRPV1 and TAS2Rs associate with sensations from sampled ethanol. Alcoholism, clinical and experimental research.

[43] Nolden AA, McGeary JE, Hayes JE (2016). Differential bitterness in capsaicin, piperine, and ethanol associates with polymorphisms in multiple bitter taste receptor genes. Physiology & behavior.

[44] Hayes JE (2011). Allelic variation in TAS2R bitter receptor genes associates with variation in sensations from and ingestive behaviors toward common bitter beverages in adults. Chemical senses.

[45] De Rosso M (2010). Chemical characterization and enological potential of Raboso varieties by study of secondary grape metabolites. Journal of agricultural and food chemistry.

[46] Carlson CS (2004). Selecting a maximally informative set of single-nucleotide polymorphisms for association analyses using linkage disequilibrium. American journal of human genetics.

[47] Barrett JC, Fry B, Maller J, Daly MJ (2005). Haploview: analysis and visualization of LD and haplotype maps. Bioinformatics.

[48] de Bakker PI (2005). Efficiency and power in genetic association studies. Nature genetics.

[49] Gao X, Starmer J, Martin ER (2008). A multiple testing correction method for genetic association studies using correlated single nucleotide polymorphisms. Genetic epidemiology.

[50] Boyle AP (2012). Annotation of functional variation in personal genomes using RegulomeDB. Genome research.

[51] Ward LD, Kellis M (2012). HaploReg: a resource for exploring chromatin states, conservation, and regulatory motif alterations within sets of genetically linked variants. Nucleic acids research.

[52] GTEX Consortium, G. C. The Genotype-Tissue Expression (GTEx) project. *Nature genetics* **45**, 580–585, doi:10.1038/ng.2653 (2013). 10.1038/ng.2653 PMC4010069 23715323

