# Supplementary material for: Association between taste receptor (TAS) genes and the perception of wine characteristics
Source: Sci Rep. 2017 Aug 23;7:9239. doi: 10.1038/s41598-017-08946-3 (PMC5569080; doi:10.1038/s41598-017-08946-3)
Supplement: Supplementary file 1 — Supplementary tables [file 41598_2017_8946_MOESM1_ESM.pdf]

**Supplemantary Tables**

**Title of the manuscript:** Association between taste receptor (TAS) genes and the perception of wine characteristics

**Author list:** Maura Carrai Daniele Campa, Pavel Vodicka, Riccardo Flamini, Irene Martelli, Jana Slyskova, Katerina Jiraskova, Alexandra Rejhova, Sona Vodenkova, Federico Canzian, Alberto Bertelli, Antonio Dalla Vedova, Luigi Bavaresco, Ludmila Vodickova, Roberto Barale

**Supplementary table S1. Association of all the SNPs with PROP sensitivity**

| Variabile indipendente | Livello (se 0 unico livello) | Coef. | P>\|z\| | [95% Conf Interval] |
|---|---|---|---|---|
| CA6_rs2274333 | Mm Vs MM | -0.020 | 0.923 | (-0,4269009; 0,3868488) |
| CA6_rs2274333 | mm Vs MM | -0.195 | 0.557 | (-0,8453703; 0,4553909) |
| GNAT3_rs7792845 | Mm Vs MM | 0.166 | 0.346 | (-0,179418; 0,5114185) |
| GNAT3_rs7792845 | mm Vs MM | -0.246 | 0.309 | (-0,7209397; 0,2283362) |
| TAS1R2_rs35874116 | Mm Vs MM | -0.043 | 0.799 | (-0,3736597; 0,2879028) |
| TAS1R2_rs35874116 | mm Vs MM | 0.304 | 0.279 | (-0,246117; 0,8540741) |
| TAS1R2_rs4920566 | Mm Vs MM | 0.155 | 0.466 | (-0,2613635; 0,5704173) |
| TAS1R2_rs4920566 | mm Vs MM | 0.334 | 0.139 | (-0,1083795; 0,7765003) |
| TAS1R3_rs35744813 | Mm Vs MM | 0.070 | 0.249 | (-0,416956; 0,5576089) |
| TAS2R1_rs2234233 | Mm Vs MM | -0.065 | 0.723 | (-0,4260983; 0,2956269) |
| TAS2R1_rs2234233 | mm Vs MM | -0.200 | 0.646 | (-1,051481; 0,651834) |
| TAS2R1_rs41467 | Mm Vs MM | -0.027 | 0.874 | (-0,363959; 0,3094399) |
| TAS2R1_rs41467 | mm Vs MM | 0.124 | 0.601 | (-0,3415453; 0,5901937) |
| TAS2R14_rs11610105 | Mm Vs MM | 0.067 | 0.687 | (-0,2579887; 0,3912677) |
| TAS2R14_rs11610105 | mm Vs MM | 0.246 | 0.454 | (-0,398221; 0,8907656) |
| TAS2R16_rs6466849 | Mm Vs MM | -0.102 | 0.567 | (-0,4529785; 0,248324) |
| TAS2R16_rs6466849 | mm Vs MM | 0.561 | 0.326 | (-0,5591528; 1,681884) |
| TAS2R16_rs860170 | Mm Vs MM | -0.176 | 0.294 | (-0,5050025; 0,1526425) |
| TAS2R16_rs860170 | mm Vs MM | -0.002 | 0.993 | (-0,5431011; 0,5382848) |
| TAS2R16_rs978739 | Mm Vs MM | -0.300 | 0.076 | (-0,6316194; 0,0317195) |
| TAS2R16_rs978739 | mm Vs MM | -0.058 | 0.832 | (-0,5901395; 0,4746832) |
| TAS2R19_rs12313469 | Mm Vs MM | 0.263 | 0.186 | (-0,1269921; 0,6537945) |
| TAS2R19_rs12313469 | mm Vs MM | 0.241 | 0.302 | (-0,2168097; 0,6995747) |
| TAS2R20_rs10772408 | Mm Vs MM | -0.048 | 0.785 | (-0,3908963; 0,2955698) |
| TAS2R20_rs10772408 | mm Vs MM | -0.059 | 0.810 | (-0,5375968; 0,4199933) |
| TAS2R30_rs2599404 | Mm Vs MM | 0.039 | 0.849 | (-0,3617732; 0,4398025) |
| TAS2R30_rs2599404 | mm Vs MM | 0.044 | 0.851 | (-0,4158175; 0,5041729) |
| TAS2R31_rs10845296 | Mm Vs MM | -0.035 | 0.847 | (-0,3933804; 0,3226584) |
| TAS2R31_rs10845296 | mm Vs MM | 0.393 | 0.405 | (-0,532909; 1,319214) |
| TAS2R38_rs10246939 | Mm Vs MM | 0.368 | 0.053 | (-0,0039964; 0,7390507) |
| TAS2R38_rs10246939 | mm Vs MM | 1.710 | 0.000 | (1,296952; 2,124031) |
| TAS2R38_rs1726866 | Mm Vs MM | 0.366 | 0.054 | (-0,005835; 0,7371022) |
| TAS2R38_rs1726866 | mm Vs MM | 1.720 | 0.000 | (1,306599; 2,134023) |
| TAS2R38_rs713598 | Mm Vs MM | 0.358 | 0.079 | (-0,0408327; 0,7560625) |
| TAS2R38_rs713598 | mm Vs MM | 1.633 | 0.000 | (1,210333; 2,055768) |
| TAS2R39_rs4726600 | Mm Vs MM | -0.189 | 0.288 | (-0,5363169; 0,1592148) |
| TAS2R39_rs4726600 | mm Vs MM | 0.145 | 0.684 | (-0,5518018; 0,8411787) |
| TAS2R40_rs534126 | Mm Vs MM | -0.122 | 0.462 | (-0,448158; 0,2037637) |
| TAS2R40_rs534126 | mm Vs MM | 0.075 | 0.785 | (-0,4615715; 0,6107118) |
| TAS2R41_rs10278721 | Mm Vs MM | -0.091 | 0.593 | (-0,4246336; 0,2425518) |
| TAS2R41_rs10278721 | mm Vs MM | -0.465 | 0.103 | (-1,023853; 0,0934816) |
| TAS2R42_rs5020531 | Mm Vs MM | -0.004 | 0.983 | (-0,3424817; 0,3351725) |

| | | | | |
|---|---|---|---|---|
| TAS2R42_rs5020531 | mm Vs MM | 0.096 | 0.708 | (-0,4041749; 0,5955355) |
| TAS2R46_rs2708381 | Mm Vs MM | 0.331 | 0.065 | (-0,0208535; 0,6820387) |
| TAS2R46_rs2708381 | mm Vs MM | -0.127 | 0.757 | (-0,9317792; 0,6775085) |
| TAS2R5_rs2227264 | Mm Vs MM | -0.635 | 0.001 | (-1,021399; -0,2476244) |
| TAS2R5_rs2227264 | mm Vs MM | -0.931 | 0.000 | (-1,363882; -0,4971271) |
| TAS2R50_rs1376251 | Mm Vs MM | -0.039 | 0.824 | (-0,3876064; 0,3087661) |
| TAS2R50_rs1376251 | mm Vs MM | 0.010 | 0.996 | (-0,545409; 0,5427206) |
| TAS2R50_rs7136962 | Mm Vs MM | 0.203 | 0.228 | (-0,1271773; 0,5340109) |
| TAS2R50_rs7136962 | mm Vs MM | -0.060 | 0.875 | (-0,8096406; 0,6894947) |
| TAS2R60_rs4595035 | Mm Vs MM | 0.046 | 0.788 | (-0,2891064; 0,3811795) |
| TAS2R60_rs4595035 | mm Vs MM | -0.059 | 0.814 | (-0,552465; 0,4340965) |
| TAS2R7_rs2588350 | Mm Vs MM | 0.207 | 0.259 | (-0,1524614; 0,5659731) |
| TAS2R7_rs2588350 | mm Vs MM | 0.355 | 0.221 | (-0,2140503; 0,9247068) |
| TAS2R9_rs3741845 | Mm Vs MM | 0.116 | 0.524 | (-0,2409942; 0,4732343) |
| TAS2R9_rs3741845 | mm Vs MM | 0.038 | 0.883 | (-0,4726842; 0,5491657) |

**Mn Vs MM defines the analysis of heterozygous versus common homozygous**

**mm Vs MM defines the analysis of rare homozygous versus common homozygous**

**Supplementary table S2. Associations between the selected polymorphic variants and wine bitterness.**

| Chr. | Gene | SNP | Model | Czech Republic | | | Italy | | | Czech Republic and Italy | | |
|---|---|---|---|---|---|---|---|---|---|---|---|---|
| | | | | Coef. | 95% CI | P-value | Coef. | 95% CI | P-value | Coef. | 95% CI | P-value |
| **Chr.1** | | | | | | | | | | | | |
| | *TAS1R3* | rs35744813 | T/C vs C/C | -0.29 | (-1.31 ; 0.74) | 0.52 | -0.72 | (-1.96 ; 0.51) | 0.63 | -0.43 | (-1.23 ; 0.36) | 0.41 |
| | *CA6* | rs2274333 | G/A vs A/A | 0.07 | (-0.75 ; 0.89) | 0.86 | n.a. | n.a. | n.a. | n.a. | n.a. | n.a. |
| | | | G/G vs /A/A | -0.53 | (-1.74 ; 0.68) | 0.39 | n.a. | n.a. | n.a. | n.a. | n.a. | n.a. |
| | *TAS1R2* | rs4920566 | G/A vs G/G | 0.40 | (-0.40 ; 1.21) | 0.33 | n.a. | n.a. | n.a. | n.a. | n.a.) | n.a. |
| | | | A/A vs G/G | -0.66 | (-1.55 ; 0.22) | 0.14 | n.a. | n.a. | n.a. | n.a. | n.a. | n.a. |
| | | rs35874116 | G/A vs A/A | 0.11 | (-0.60 ; 0.82) | 0.76 | 0.41 | (-0.42 ; 1.23) | 0.34 | 0.19 | (-0.35 ; 0.73) | 0.50 |
| | | | G/G vs A/A | -0.18 | (-1.41 ; 1.06) | 0.78 | -0.45 | (-1.75 ; 0.84) | 0.49 | -0.32 | (-1.22 ; 0.58) | 0.49 |
| **Chr.5** | | | | | | | | | | | | |
| | *TAS1R1* | rs41467 | T/G vs G/G | -0.03 | (-0.76  0.71) | 0.94 | -0.21 | (-1.00 ; 0.58) | 0.60 | -0.17 | (-0.70 ; 0.37) | 0.55 |
| | | | T/T vs G/G | -0.25 | (-1.22 ; 0.73) | 0.62 | -0.50 | (-1.63 ; 0.64) | 0.39 | -0.38 | (-1.12 ; 0.36) | 0.31 |
| **Chr.7** | | | | | | | | | | | | |
| | *GNAT3* | rs7792845 | G/A vs G/G | 0.22 | (-0.49 ; 0.94) | 0.54 | -0.08 | (-0.94 ; 0.77) | 0.85 | 0.16 | (-0.40 ; 0.71) | 0.58 |
| | | | A/A vs G/G | 1.19 | (-0.03 ; 2.40) | 0.06 | -0.87 | (-1.88 ; 0.13) | 0.09 | -0.14 | (-0.90 ; 0.63) | 0.73 |
| | *TAS2R16* | rs6466849 | A/G vs G/G | 0.33 | (-0.40 ; 1.05) | 0.38 | -0.51 | (-1.35 ; 0.33) | 0.23 | -0.05 | (-0.61 ; 0.50) | 0.85 |
| | | | A/A vs G/G | -0.01 | (-1.88 ; 1.86) | 0.99 | -3.81 | (-9.22 ; 1.61) | 0.17 | -0.53 | (-2.30 ; 1.24) | 0.56 |
| | | rs860170 | G/A vs /A/A | -0.34 | (-1.06 ; 0.39) | 0.36 | 0.39 | (-0.39 ; 1.17) | 0.33 | 0.06 | (-0.47 ; 0.6) | 0.82 |
| | | | G/G vs A/A | 0.06 | (-0.98 ; 1.10) | 0.91 | 0.81 | (-0.72 ; 2.34) | 0.30 | 0.38 | (-0.49 ; 1.24) | 0.40 |
| | | rs978739 | G/A vs A/A | 0.11 | (-0.60 ; 0.82) | 0.76 | -0.13 | (-0.92 ; 0.65) | 0.74 | -0.04 | (-0.57 ; 0.48) | 0.87 |
| | | | G/G vs A/A | 0.42 | (-0.64 ; 1.48) | 0.44 | 0.24 | (-1.17 ; 1.64) | 0.74 | 0.38 | (-0.46 ; 1.22) | 0.38 |
| | *TAS2R5* | rs2227264 | T/G vs G/G | -0.53 | (-1.36 ; 0.30) | 0.21 | 0.89 | (-0.03 ; 1.82) | 0.06 | 0.10 | (-0.53 ; 0.73) | 0.75 |
| | | | T/T vs G/G | -0.08 | (-1.00 ; 0.83) | 0.86 | 0.46 | (-0.59 ; 1.52) | 0.39 | 0.21 | (-0.49 ; 0.91) | 0.56 |
| | *TAS2R38* | rs10246939 | C/T vs C/C | -0.90 | (-1.78 ; -0.01) | 0.05 | -0.06 | (-0.96 ; 0.84) | 0.90 | -0.54 | (-1.17 ; 0.09) | 0.10 |
| | | | T/T vs C/C | -1.12 | (-2.11 ; -0.14) | 0.03 | -0.97 | (-1.96 ; 0.02) | 0.06 | -1.08 | (-1.78 ; -0.38) | **0.003** |
| | | rs1726866 | T/C vs C/C | -1.06 | (-1.94 ; -0.19) | 0.02 | -0.07 | (-0.97 ; 0.83) | 0.89 | -0.63 | (-1.27 ; -0.001) | 0.05 |
| | | | T/C vs C/C | -1.16 | (-2.13 ; -0.18) | 0.02 | -0.94 | (-1.94 ; 0.05) | 0.06 | -1.10 | (-1.80 ; -0.40) | **0.002** |
| | | rs713598 | G/C vs C/C | -0.71 | (-1.67 ; 0.26) | 0.15 | 0.08 | (-0.87 ; 1.03) | 0.87 | -0.34 | (-1.02 ; 0.34) | 0.33 |
| | | | G/G vs C/C | -0.91 | (-1.93 ; 0.10) | 0.08 | -0.51 | (-1.51 ; 0.50) | 0.32 | -0.75 | (-1.46 ; -0.03) | 0.04 |
| | *TAS2R39* | rs4726600 | G/A vs G/G | -0.08 | (-0.78 ; 0.62) | 0.82 | 0.14 | (-0.75 ; 1.03) | 0.76 | 0.00 | (-0.56 ; 0.55) | 0.99 |
| | | | A/A vs G/G | -0.87 | (-2.30 ; 0.56) | 0.23 | -0.64 | (-2.36 ; 1.08) | 0.47 | -0.66 | (-1.76 ; 0.45) | 0.24 |
| | *TAS2R40* | rs534126 | T/C vs C/C | -0.54 | (-1.23 ; 0.14) | 0.12 | -0.31 | (-1.09 ; 0.48) | 0.45 | -0.46 | (-0.98 ; 0.06) | 0.08 |
| | | | T/T vs C/C | 0.51 | (-0.74 ; 1.75) | 0.43 | -0.32 | (-1.50 ; 0.85) | 0.59 | 0.04 | (-0.81 ; 0.90) | 0.92 |
| | *TAS2R60* | rs4595035 | T/C vs C/C | -0.06 | (-0.77 ; 0.65) | 0.88 | -0.16 | (-0.95 ; 0.63) | 0.69 | -0.05 | (-0.59 ; 0.48) | 0.84 |
| | | | T/T vs C/C | -0.41 | (-1.49 ; 0.66) | 0.45 | -0.50 | (-1.65 ; 0.65) | 0.39 | -0.42 | (-1.21 ; 0.37) | 0.30 |

| | | | | | | | | | | | |
|---|---|---|---|---|---|---|---|---|---|---|---|
| | *TAS2R41* | rs10278721 | T/C vs C/C | 0.33 | (-0.36 ; 1.03) | 0.34 | -0.07 | (-0.89 ; 0.75) | 0.87 | 0.16 | (-0.38 ; 0.69) | 0.56 |
| | | | T/T vs C/C | -0.13 | (-1.45 ; 1.18) | 0.84 | 0.39 | (-0.80 ; 1.58) | 0.52 | 0.15 | (-0.74 ; 1.03) | 0.75 |
| **Chr.12** | | | | | | | | | | | | |
| | *TAS2R7* | rs2588350 | G/A vs G/G | 0.32 | (-0.38 ; 1.02) | 0.38 | 0.89 | (-0.11 ; 1.89) | 0.08 | 0.54 | (-0.04 ; 1.12) | 0.07 |
| | | | A/A vs G/G | 0.30 | (-1.15 ; 1.76) | 0.68 | 0.55 | (-0.66 ; 1.75) | 0.38 | 0.43 | (-0.48 ; 1.35) | 0.35 |
| | *TAS2R9* | rs3741845 | T/C vs C/C | -0.13 | (-0.87 ; 0.60) | 0.72 | 0.45 | (-0.42 ; 1.31) | 0.31 | 0.12 | (-0.45 ; 0.68) | 0.69 |
| | | | T/T vs C/C | 0.05 | (-0.91 ; 1.02) | 0.92 | 0.30 | (-1.16 ; 1.77) | 0.69 | 0.10 | (-0.71 ; 0.91) | 0.81 |
| | *TAS2R14* | rs11610105 | G/A vs G/G | 0.49 | (-0.20 ; 1.17) | 0.16 | -0.56 | (-1.35 ; 0.22) | 0.16 | 0.06 | (-0.46 ; 0.58) | 0.82 |
| | | | A/A vs G/G | 0.01 | (-1.38 ; 1.41) | 0.99 | 0.15 | (-1.33 ; 1.64) | 0.84 | -0.05 | (-1.07 ; 0.98) | 0.93 |
| | *TAS2R50* | rs1376251 | T/C vs C/C | 0.74 | (0.05 ; 1.42) | 0.04 | 0.47 | (-0.42 ; 1.36) | 0.30 | 0.69 | (0.15 ; 1.24) | 0.01 |
| | | | T/T vs C/C | -0.74 | (-1.96 ; 0.48) | 0.23 | 0.89 | (-0.30 ; 2.08) | 0.14 | 0.16 | (-0.69 ; 1.01) | 0.71 |
| | | rs7136962 | T/C vs T/T | 0.40 | (-0.29 ; 1.09) | 0.26 | -0.13 | (-0.94 ; 0.67) | 0.74 | 0.17 | (-0.36 ; 0.70) | 0.53 |
| | | | C/C vs T/T | 0.04 | (-1.42 ; 1.50) | 0.96 | 0.57 | (-1.43 ; 2.56) | 0.58 | 0.14 | (-1.05 ; 1.33) | 0.82 |
| | *TAS2R20* | rs10772408 | G/A vs G/G | -0.28 | (-1.02 ; 0.46) | 0.45 | -0.57 | (-1.36 ; 0.21) | 0.15 | -0.44 | (-0.98 ; 0.10) | 0.11 |
| | | | A/A vs G/G | -0.33 | (-1.35 ; 0.68) | 0.52 | -0.62 | (-1.77 ; 0.54) | 0.30 | -0.47 | (-1.24 ; 0.29) | 0.23 |
| | *TAS2R19* | rs12313469 | G/A vs G/G | 0.65 | (-0.26 ; 1.57) | 0.16 | -1.13 | (-1.97 ; -0.29) | 0.01 | -0.30 | (-0.93 ; 0.32) | 0.35 |
| | | | A/A vs G/G | 0.14 | (-0.89 ; 1.17) | 0.79 | -0.98 | (-2.04 ; 0.08) | 0.07 | -0.62 | (-1.36 ; 0.11) | 0.10 |
| | *TAS2R31* | rs10845296 | T/C vs T/T | 0.07 | (-0.69 ; 0.82) | 0.86 | 0.30 | (-0.58 ; 1.18) | 0.50 | 0.19 | (-0.38 ; 0.77) | 0.51 |
| | | | C/C vs T/T | -1.20 | (-3.92 ; 1.52) | 0.39 | 0.36 | (-1.50 ; 2.22) | 0.71 | -0.03 | (-1.56 ; 1.49) | 0.97 |
| | *TAS2R46* | rs2708381 | G/A vs G/G | 0.45 | (-0.31 ; 1.22) | 0.25 | -0.01 | (-0.81 ; 0.79) | 0.98 | 0.23 | (-0.33 ; 0.78) | 0.42 |
| | | | A/A vs G/G | -0.15 | (-1.77 ; 1.47) | 0.86 | -0.09 | (-2.08 ; 1.90) | 0.93 | -0.22 | (-1.48 ; 1.04) | 0.74 |
| | *TAS2R30* | rs2599404 | T/G vs T/T | 0.70 | (-0.19 ; 1.59) | 0.12 | -0.67 | (-1.58 ; 0.25) | 0.16 | 0.04 | (-0.60 ; 0.67) | 0.91 |
| | | | G/G vs T/T | 0.30 | (-0.70 ; 1.30) | 0.55 | -0.77 | (-1.86 ; 0.33) | 0.17 | -0.32 | (-1.05 ; 0.42) | 0.40 |
| | *TAS2R42* | rs5020531 | T/C vs C/C | 0.08 | (-0.66 ; 0.81) | 0.84 | 0.00 | (-0.81 ; 0.81) | 1.00 | 0.12 | (-0.42 ; 0.67) | 0.66 |
| | | | T/C vs C/C | -0.21 | (-1.21 ; 0.80) | 0.69 | 0.05 | (-1.27 ; 1.36) | 0.94 | -0.14 | (-0.94 ; 0.66) | 0.73 |

**Supplementary table S3. Associations between the selected polymorphic variants and wine sourness.**

| Chr. | Gene | SNP | Model | Czech Republic | | | Italy | | | Czech Republic and Italy | | |
|---|---|---|---|---|---|---|---|---|---|---|---|---|
| | | | | Coef. | 95% CI | P-value | Coef. | 95% CI | P-value | Coef. | 95% CI | P-value |
| **Chr.1** | | | | | | | | | | | | |
| | *TAS1R3* | rs357448 | T/C vs C/C | 0.31 | (-0.70 ; 1.31) | 0.51 | -0.41 | (-1.50 ; 0.68) | 0.56 | 0.00 | (0.73 ; 0.74) | 0.38 |
| | *CA6* | rs227433 | G/A vs A/A | 0.48 | (-0.34 ; 1.30) | 0.25 | n.a. | n.a. | n.a. | n.a. | n.a. | n.a. |
| | | | G/G vs /A/A | -0.20 | (-1.44 ; 1.03) | 0.75 | n.a. | n.a. | n.a. | n.a. | n.a. | n.a. |
| | *TAS1R2* | rs492056 | G/A vs G/G | 0.17 | (-0.63 ; 0.98) | 0.68 | n.a. | n.a. | n.a. | n.a. | n.a. | n.a. |
| | | | A/A vs G/G | -0.27 | (-1.16 ; 0.61) | 0.55 | n.a. | n.a. | n.a. | n.a. | n.a. | n.a. |
| | | rs358741 | G/A vs A/A | -0.28 | (-0.97 ; 0.42) | 0.44 | -0.44 | (-1.16 ; 0.28) | 0.23 | -0.37 | (-0.87 ; 0.13) | 0.15 |
| | | | G/G vs A/A | 0.73 | (-0.48 ; 1.93) | 0.24 | -0.85 | (-1.97 ; 0.28) | 0.14 | -0.04 | (-0.87 ; 0.78) | 0.92 |
| **Chr.5** | | | | | | | | | | | | |
| | *TAS1R1* | rs41467 | T/G vs G/G | -0.27 | (-0.99 ; 0.46) | 0.47 | -0.23 | (-0.93 ; 0.48) | 0.53 | -0.26 | (-0.77 ; 0.24) | 0.30 |
| | | | T/T vs G/G | -0.81 | (-1.76 ; 0.15) | 0.10 | 0.05 | (-0.97 ; 1.06) | 0.93 | -0.46 | (-1.16 ; 0.23) | 0.19 |
| **Chr.7** | | | | | | | | | | | | |
| | *GNAT3* | rs779284 | G/A vs G/G | 0.29 | (-0.42 ; 0.99) | 0.42 | -0.43 | (-1.20 ; 0.34) | 0.28 | 0.02 | (-0.50 ; 0.54) | 0.95 |
| | | | A/A vs G/G | 0.64 | (-0.56 ; 1.84) | 0.30 | -0.47 | (-1.38 ; 0.43) | 0.30 | 0.02 | (-0.69 ; 0.73) | 0.95 |
| | *TAS2R16* | rs646684 | A/G vs G/G | 1.20 | (0.50 ; 1.91) | 0.001 | 0.61 | (-0.14 ; 1.35) | 0.11 | 0.94 | (0.43 ; 1.46) | 0.0003 |
| | | | A/A vs G/G | -0.75 | (-2.56 ; 1.05) | 0.41 | -5.11 | (-9.93 ; 0.28) | 0.04 | -1.30 | (-2.94 ; 0.34) | 0.12 |
| | | rs860170 | G/A vs /A/A | 0.10 | (-0.61 ; 0.82) | 0.78 | 0.07 | (-0.61 ; 0.75) | 0.84 | 0.10 | (-0.39 ; 0.60) | 0.68 |
| | | | G/G vs A/A | 0.24 | (-0.80 ; 1.27) | 0.65 | 0.25 | (-1.08 ; 1.58) | 0.71 | 0.24 | (-0.56 ; 1.05) | 0.55 |
| | | rs978739 | G/A vs A/A | 0.29 | (-0.41 ; 0.99) | 0.42 | 0.59 | (-0.12 ; 1.29) | 0.10 | 0.40 | (-0.09 ; 0.90) | 0.11 |
| | | | G/G vs A/A | 0.46 | (-0.59 ; 1.50) | 0.39 | 0.17 | (-1.09 ; 1.42) | 0.80 | 0.40 | (-0.39 ; 1.19) | 0.33 |
| | *TAS2R5* | rs222726 | T/G vs G/G | -0.52 | (-1.35 ; 0.32) | 0.22 | 0.28 | (-0.56 ; 1.11) | 0.52 | -0.17 | (-0.76 ; 0.43) | 0.58 |
| | | | T/T vs G/G | -0.90 | (-1.82 ; 0.02) | 0.06 | -0.04 | (-0.99 ; 0.91) | 0.93 | -0.49 | (-1.15 ; 0.17) | 0.15 |
| | *TAS2R38* | rs102469 | C/T vs C/C | 0.49 | (-0.39 ; 1.37) | 0.27 | 0.60 | (-0.21 ; 1.40) | 0.15 | 0.50 | (-0.10 ; 1.10) | 0.10 |
| | | | T/T vs C/C | 0.64 | (-0.34 ; 1.62) | 0.20 | -0.02 | (-0.91 ; 0.87) | 0.96 | 0.30 | (-0.36 ; 0.96) | 0.37 |
| | | rs172686 | T/C vs C/C | 0.42 | (-0.46 ; 1.31) | 0.35 | 0.59 | (-0.21 ; 1.40) | 0.15 | 0.46 | (-0.14 ; 1.06) | 0.13 |
| | | | T/C vs C/C | 0.59 | (-0.39 ; 1.58) | 0.24 | -0.03 | (-0.92 ; 0.86) | 0.94 | 0.27 | (-0.40 ; 0.93) | 0.43 |
| | | rs713598 | G/C vs C/C | -0.08 | (-1.03 ; 0.87) | 0.87 | 0.13 | (-0.72 ; 0.98) | 0.76 | -0.02 | (-0.66 ; 0.62) | 0.95 |
| | | | G/G vs C/C | 0.56 | (-0.45 ; 1.57) | 0.28 | -0.07 | (-0.97 ; 0.82) | 0.87 | 0.24 | (-0.44 ; 0.91) | 0.49 |
| | *TAS2R39* | rs472660 | G/A vs G/G | 0.17 | (-0.52 ; 0.86) | 0.64 | -0.21 | (-0.99 ; 0.58) | 0.60 | 0.03 | (-0.49 ; 0.54) | 0.92 |
| | | | A/A vs G/G | -0.57 | (-1.98 ; 0.84) | 0.43 | 0.06 | (-1.46 ; 1.57) | 0.94 | -0.28 | (-1.31 ; 0.75) | 0.59 |
| | *TAS2R40* | rs534126 | T/C vs C/C | 0.35 | (-0.32 ; 1.02) | 0.31 | -0.36 | (-1.06 ; 0.34) | 0.31 | 0.04 | (-0.45 ; 0.52) | 0.88 |
| | | | T/T vs C/C | 1.44 | (0.19 ; 2.68) | 0.02 | 0.06 | (-0.98 ; 1.11) | 0.91 | 0.73 | (-0.08 ; 1.54) | 0.08 |
| | *TAS2R60* | rs459503 | T/C vs C/C | -0.46 | (-1.16 ; 0.23) | 0.19 | 0.12 | (-0.59 ; 0.82) | 0.74 | -0.19 | (-0.69 ; 0.31) | 0.45 |
| | | | T/T vs C/C | -0.30 | (-1.36 ; 0.76) | 0.58 | 0.31 | (-0.71 ; 1.33) | 0.56 | -0.02 | (-0.75 ; 0.72) | 0.97 |

| | | | | | | | | | | | | |
|---|---|---|---|---|---|---|---|---|---|---|---|---|
| | *TAS2R41* | rs102787 | T/C vs C/C | -0.14 | (-0.81 ; 0.54) | 0.70 | -0.13 | (-0.86 ; 0.61) | 0.73 | -0.14 | (-0.64 ; 0.36) | 0.58 |
| | | | T/T vs C/C | 0.59 | (-0.69 ; 1.88) | 0.37 | -0.05 | (-1.11 ; 1.01) | 0.93 | 0.22 | (-0.60 ; 1.05) | 0.60 |
| **Chr.12** | | | | | | | | | | | | |
| | *TAS2R7* | rs258835 | G/A vs G/G | 0.16 | (-0.53 ; 0.85) | 0.65 | 0.45 | (-0.44 ; 1.34) | 0.32 | 0.28 | (-0.26 ; 0.83) | 0.31 |
| | | | A/A vs G/G | 1.45 | (0.02 ; 2.89) | 0.05 | 0.33 | (-0.74 ; 1.39) | 0.55 | 0.70 | (-0.15 ; 1.56) | 0.11 |
| | *TAS2R9* | rs374184 | T/C vs C/C | 0.35 | (-0.37 ; 1.07) | 0.33 | -0.24 | (-1.03 ; 0.55) | 0.55 | 0.12 | (-0.42 ; 0.65) | 0.67 |
| | | | T/T vs C/C | 0.78 | (-0.17 ; 1.72) | 0.11 | -1.30 | (-2.63 ; 0.04) | 0.06 | 0.15 | (-0.62 ; 0.91) | 0.71 |
| | *TAS2R14* | rs116101 | G/A vs G/G | 0.21 | (-0.47 ; 0.88) | 0.55 | -0.27 | (-0.97 ; 0.43) | 0.45 | 0.00 | (-0.48 ; 0.49) | 0.99 |
| | | | A/A vs G/G | 0.28 | (-1.09 ; 1.66) | 0.69 | -0.66 | (-1.98 ; 0.67) | 0.33 | -0.20 | (-1.16 ; 0.76) | 0.68 |
| | *TAS2R50* | rs137625 | T/C vs C/C | 0.29 | (-0.39 ; 0.97) | 0.41 | 0.25 | (-0.54 ; 1.04) | 0.54 | 0.30 | (-0.21 ; 0.81) | 0.25 |
| | | | T/T vs C/C | -0.28 | (-1.51 ; 0.96) | 0.66 | 0.59 | (-0.47 ; 1.65) | 0.28 | 0.20 | (-0.61 ; 1.01) | 0.62 |
| | | rs713696 | T/C vs T/T | 0.28 | (-0.40 ; 0.97) | 0.42 | -0.07 | (-0.79 ; 0.65) | 0.85 | 0.13 | (-0.37 ; 0.62) | 0.61 |
| | | | C/C vs T/T | 0.25 | (-1.20 ; 1.71) | 0.73 | -0.55 | (-2.33 ; 1.23) | 0.54 | -0.07 | (-1.19 ; 1.04) | 0.90 |
| | *TAS2R20* | rs107724 | G/A vs G/G | 0.02 | (-0.71 ; 0.75) | 0.96 | -0.45 | (-1.16 ; 0.25) | 0.21 | -0.20 | (-0.71 ; 0.30) | 0.43 |
| | | | A/A vs G/G | -0.58 | (-1.57 ; 0.41) | 0.25 | -0.25 | (-1.28 ; 0.78) | 0.63 | -0.46 | (-1.18 ; 0.25) | 0.20 |
| | *TAS2R19* | rs123134 | G/A vs G/G | 1.04 | (0.14 ; 1.94) | 0.02 | -0.47 | (-1.23 ; 0.28) | 0.22 | 0.23 | (-0.36 ; 0.81) | 0.45 |
| | | | A/A vs G/G | -0.02 | (-1.03 ; 0.99) | 0.97 | -0.81 | (-1.76 ; 0.15) | 0.10 | -0.58 | (-1.26 ; 0.11) | 0.10 |
| | *TAS2R31* | rs108452 | T/C vs T/T | 0.44 | (-0.31 ; 1.18) | 0.25 | 0.06 | (-0.72 ; 0.84) | 0.89 | 0.28 | (-0.26 ; 0.82) | 0.31 |
| | | | C/C vs T/T | 0.91 | (-1.79 ; 3.61) | 0.51 | 0.07 | (-1.59 ; 1.72) | 0.94 | 0.40 | (-1.03 ; 1.83) | 0.58 |
| | *TAS2R46* | rs270838 | G/A vs G/G | 0.49 | (-0.28 ; 1.25) | 0.21 | -0.34 | (-1.05 ; 0.38) | 0.36 | 0.09 | (-0.44 ; 0.61) | 0.74 |
| | | | A/A vs G/G | 0.01 | (-1.61 ; 1.62) | 0.99 | 0.45 | (-1.34 ; 2.23) | 0.63 | 0.10 | (-1.09 ; 1.29) | 0.87 |
| | *TAS2R30* | rs259940 | T/G vs T/T | 0.68 | (-0.19 ; 1.56) | 0.13 | -0.52 | (-1.34 ; 0.29) | 0.21 | 0.09 | (-0.51 ; 0.69) | 0.76 |
| | | | G/G vs T/T | -0.35 | (-1.33 ; 0.64) | 0.49 | -1.24 | (-2.22 ; 0.27) | 0.01 | -0.85 | (-1.54 ; 0.15) | 0.02 |
| | *TAS2R42* | rs502053 | T/C vs C/C | 0.19 | (-0.53 ; 0.91) | 0.60 | -0.26 | (-0.97 ; 0.46) | 0.48 | 0.01 | (-0.49 ; 0.52) | 0.96 |
| | | | T/C vs C/C | 0.82 | (-0.17 ; 1.8) | 0.10 | 0.41 | (-0.75 ; 1.57) | 0.49 | 0.63 | (-0.11 ; 1.38) | 0.10 |

**Supplementary table S4. Associations between the selected polymorphic variants and e astringency.**

| Chr. | Gene | SNP | Model | Czech Republic | | | Italy | | | Czech Republic and Italy | | |
|---|---|---|---|---|---|---|---|---|---|---|---|---|
| | | | | Coef. | 95% CI | P-value | Coef. | 95% CI | P-value | Coef. | 95% CI | P-value |
| Chr.1 | | | | | | | | | | | | |
| | *TAS1R3* | rs3574481 | T/C vs C/C | -0.22 | (-1.09 ; 0.66) | 0.45 | -0.19 | (-1.33 ; 0.95) | 0.58 | -0.14 | (-0.84 ; 0.57) | 0.36 |
| | *CA6* | rs2274333 | G/A vs A/A | 0.12 | (-0.57 ; 0.80) | 0.74 | n.a. | n.a. | n.a. | n.a. | n.a. | n.a. |
| | | | G/G vs /A/A | -0.59 | (-1.6 ; 0.430) | 0.26 | n.a. | n.a. | n.a. | n.a. | n.a. | n.a. |
| | *TAS1R2* | rs4920566 | G/A vs G/G | 0.29 | (-0.38 ; 0.96) | 0.40 | n.a. | n.a. | n.a. | n.a. | n.a. | n.a. |
| | | | A/A vs G/G | -1.24 | (-1.97 ; -0.50) | **0.001** | n.a. | n.a. | n.a. | n.a. | n.a. | n.a. |
| | | rs3587411 | G/A vs A/A | -0.31 | (-0.91 ; 0.30) | 0.32 | -0.08 | (-0.83 ; 0.66) | 0.83 | -0.24 | (-0.71 ; 0.24) | 0.33 |
| | | | G/G vs A/A | -0.08 | (-1.14 ; 0.98) | 0.88 | 0.80 | (-0.37 ; 1.96) | 0.18 | 0.39 | (-0.40 ; 1.17) | 0.34 |
| Chr.5 | | | | | | | | | | | | |
| | *TAS1R1* | rs41467 | T/G vs G/G | -0.20 | (-0.82 ; 0.42) | 0.53 | 0.37 | (-0.36 ; 1.10) | 0.32 | 0.04 | (-0.43 ; 0.52) | 0.86 |
| | | | T/T vs G/G | 0.34 | (-0.49 ; 1.16) | 0.42 | 0.14 | (-0.91 ; 1.19) | 0.79 | 0.25 | (-0.41 ; 0.91) | 0.46 |
| Chr.7 | | | | | | | | | | | | |
| | *GNAT3* | rs7792845 | G/A vs G/G | -0.17 | (-0.77 ; 0.44) | 0.59 | 0.16 | (-0.63 ; 0.96) | 0.69 | 0.03 | (-0.46 ; 0.52) | 0.90 |
| | | | A/A vs G/G | -0.19 | (-1.22 ; 0.85) | 0.72 | -0.26 | (-1.18 ; 0.67) | 0.59 | -0.25 | (-0.92 ; 0.42) | 0.47 |
| | *TAS2R16* | rs6466849 | A/G vs G/G | 0.44 | (-0.17 ; 1.06) | 0.16 | 0.55 | (-0.23 ; 1.33) | 0.17 | 0.49 | (0.002 ; 0.97) | 0.05 |
| | | | A/A vs G/G | 0.00 | (-1.58 ; 1.59) | 1.00 | -2.57 | (-7.60 ; 2.46) | 0.32 | -0.15 | (-1.71 ; 1.41) | 0.85 |
| | | rs860170 | G/A vs /A/A | -0.22 | (-0.83 ; 0.40) | 0.49 | 0.60 | (-0.11 ; 1.31) | 0.10 | 0.19 | (-0.28 ; 0.66) | 0.43 |
| | | | G/G vs A/A | -0.58 | (-1.46 ; 0.31) | 0.20 | -0.63 | (-2.02 ; 0.76) | 0.38 | -0.51 | (-1.27 ; 0.25) | 0.19 |
| | | rs978739 | G/A vs A/A | 0.52 | (-0.08 ; 1.12) | 0.09 | 0.33 | (-0.41 ; 1.06) | 0.39 | 0.41 | (-0.06 ; 0.88) | 0.09 |
| | | | G/G vs A/A | 0.74 | (-0.16 ; 1.64) | 0.11 | -0.40 | (-1.72 ; 0.92) | 0.55 | 0.44 | (-0.31 ; 1.19) | 0.25 |
| | *TAS2R5* | rs2227264 | T/G vs G/G | -0.44 | (-1.16 ; 0.27) | 0.23 | 0.58 | (-0.27 ; 1.44) | 0.18 | 0.03 | (-0.53 ; 0.59) | 0.92 |
| | | | T/T vs G/G | 0.05 | (-0.74 ; 0.84) | 0.90 | 0.14 | (-0.84 ; 1.12) | 0.78 | 0.17 | (-0.45 ; 0.80) | 0.59 |
| | *TAS2R38* | rs1024693 | C/T vs C/C | -0.51 | (-1.27 ; 0.25) | 0.19 | -0.18 | (-1.01 ; 0.66) | 0.68 | -0.39 | (-0.96 ; 0.17) | 0.18 |
| | | | T/T vs C/C | -0.19 | (-1.04 ; 0.65) | 0.65 | -0.72 | (-1.64 ; 0.20) | 0.13 | -0.47 | (-1.10 ; 0.15) | 0.14 |
| | | rs1726866 | T/C vs C/C | -0.57 | (-1.33 ; 0.19) | 0.14 | -0.16 | (-0.99 ; 0.67) | 0.70 | -0.41 | (-0.98 ; 0.15) | 0.15 |
| | | | T/C vs C/C | -0.27 | (-1.11 ; 0.58) | 0.54 | -0.68 | (-1.60 ; 0.24) | 0.15 | -0.50 | (-1.13 ; 0.13) | 0.12 |
| | | rs713598 | G/C vs C/C | -0.18 | (-1.01 ; 0.64) | 0.66 | -0.14 | (-1.02 ; 0.74) | 0.75 | -0.19 | (-0.79 ; 0.42) | 0.54 |
| | | | G/G vs C/C | 0.06 | (-0.81 ; 0.94) | 0.89 | -0.38 | (-1.30 ; 0.55) | 0.43 | -0.18 | (-0.81 ; 0.46) | 0.59 |
| | *TAS2R39* | rs4726600 | G/A vs G/G | 0.40 | (-0.19 ; 0.99) | 0.19 | -0.27 | (-1.07 ; 0.54) | 0.51 | 0.13 | (-0.36 ; 0.62) | 0.60 |
| | | | A/A vs G/G | 0.57 | (-0.65 ; 1.78) | 0.36 | -0.55 | (-2.11 ; 1.01) | 0.49 | 0.21 | (-0.76 ; 1.18) | 0.67 |
| | *TAS2R40* | rs534126 | T/C vs C/C | -0.41 | (-0.99 ; 0.17) | 0.16 | 0.13 | (-0.59 ; 0.85) | 0.73 | -0.20 | (-0.66 ; 0.25) | 0.38 |
| | | | T/T vs C/C | 0.56 | (-0.50 ; 1.62) | 0.30 | 0.38 | (-0.70 ; 1.46) | 0.49 | 0.46 | (-0.29 ; 1.22) | 0.23 |
| | *TAS2R60* | rs4595035 | T/C vs C/C | -0.46 | (-1.06 ; 0.14) | 0.13 | 0.37 | (-0.35 ; 1.09) | 0.31 | -0.06 | (-0.53 ; 0.41) | 0.80 |
| | | | T/T vs C/C | -0.13 | (-1.04 ; 0.78) | 0.78 | 0.37 | (-0.67 ; 1.41) | 0.49 | 0.10 | (-0.59 ; 0.79) | 0.78 |
| | *TAS2R41* | rs1027872 | T/C vs C/C | -0.05 | (-0.64 ; 0.54) | 0.86 | -0.31 | (-1.07 ; 0.45) | 0.43 | -0.19 | (-0.67 ; 0.28) | 0.42 |

| | | | | | | | | | | | |
|---|---|---|---|---|---|---|---|---|---|---|---|
| | | T/T vs C/C | -0.35 | (-1.47 ; 0.77) | 0.54 | 0.11 | (-0.99 ; 1.22) | 0.84 | -0.10 | (-0.89 ; 0.69) | 0.81 |
| **Chr.12** | | | | | | | | | | | |
| *TAS2R7* | rs2588350 | G/A vs G/G | 0.62 | (0.03 ; 1.20) | 0.04 | 0.70 | (-0.20 ; 1.60) | 0.13 | 0.68 | (0.18 ; 1.18) | 0.01 |
| | | A/A vs G/G | 1.40 | (0.18 ; 2.62) | 0.02 | 0.75 | (-0.34 ; 1.84) | 0.18 | 0.99 | (0.20 ; 1.78) | 0.01 |
| *TAS2R9* | rs3741845 | T/C vs C/C | 0.39 | (-0.23 ; 1.01) | 0.22 | -0.05 | (-0.87 ; 0.77) | 0.91 | 0.23 | (-0.28 ; 0.73) | 0.38 |
| | | T/T vs C/C | 0.35 | (-0.47 ; 1.17) | 0.40 | -0.19 | (-1.57 ; 1.19) | 0.79 | 0.13 | (-0.59 ; 0.84) | 0.73 |
| *TAS2R14* | rs1161010 | G/A vs G/G | 0.29 | (-0.29 ; 0.87) | 0.33 | 0.07 | (-0.65 ; 0.79) | 0.85 | 0.24 | (-0.22 ; 0.69) | 0.31 |
| | | A/A vs G/G | -0.53 | (-1.71 ; 0.66) | 0.38 | 0.00 | (-1.37 ; 1.37) | 1.00 | -0.33 | (-1.24 ; 0.57) | 0.47 |
| *TAS2R50* | rs1376251 | T/C vs C/C | -0.05 | (-0.64 ; 0.54) | 0.87 | 0.39 | (-0.42 ; 1.20) | 0.35 | 0.14 | (-0.34 ; 0.62) | 0.56 |
| | | T/T vs C/C | -0.93 | (-1.97 ; 0.12) | 0.08 | 0.08 | (-1.01 ; 1.17) | 0.88 | -0.46 | (-1.21 ; 0.29) | 0.23 |
| | rs7136962 | T/C vs T/T | 0.20 | (-0.39 ; 0.79) | 0.51 | -0.18 | (-0.91 ; 0.56) | 0.64 | 0.06 | (-0.41 ; 0.53) | 0.80 |
| | | C/C vs T/T | -0.53 | (-1.78 ; 0.72) | 0.41 | 0.29 | (-1.55 ; 2.12) | 0.76 | -0.26 | (-1.31 ; 0.8) | 0.63 |
| *TAS2R20* | rs1077240 | G/A vs G/G | -0.12 | (-0.75 ; 0.50) | 0.70 | -0.32 | (-1.04 ; 0.41) | 0.39 | -0.23 | (-0.71 ; 0.25) | 0.35 |
| | | A/A vs G/G | 0.73 | (-0.12 ; 1.58) | 0.09 | 0.45 | (-0.61 ; 1.51) | 0.41 | 0.58 | (-0.09 ; 1.25) | 0.09 |
| *TAS2R19* | rs1231346 | G/A vs G/G | 0.56 | (-0.22 ; 1.34) | 0.16 | -0.27 | (-1.05 ; 0.50) | 0.49 | 0.11 | (-0.44 ; 0.66) | 0.69 |
| | | A/A vs G/G | -0.03 | (-0.90 ; 0.85) | 0.95 | -0.92 | (-1.90 ; 0.05) | 0.06 | -0.52 | (-1.17 ; 0.12) | 0.11 |
| *TAS2R31* | rs1084529 | T/C vs T/T | 0.53 | (-0.11 ; 1.18) | 0.10 | 0.26 | (-0.53 ; 1.05) | 0.52 | 0.40 | (-0.10 ; 0.91) | 0.12 |
| | | C/C vs T/T | 1.50 | (-0.83 ; 3.83) | 0.21 | 0.44 | (-1.23 ; 2.12) | 0.61 | 0.87 | (-0.47 ; 2.21) | 0.20 |
| *TAS2R46* | rs2708381 | G/A vs G/G | 0.34 | (-0.31 ; 0.98) | 0.31 | -0.20 | (-0.93 ; 0.54) | 0.60 | 0.10 | (-0.39 ; 0.59) | 0.68 |
| | | A/A vs G/G | -0.75 | (-2.12 ; 0.62) | 0.29 | 0.05 | (-1.78 ; 1.87) | 0.96 | -0.47 | (-1.58 ; 0.65) | 0.41 |
| *TAS2R30* | rs2599404 | T/G vs T/T | 0.61 | (-0.15 ; 1.36) | 0.12 | -0.08 | (-0.92 ; 0.76) | 0.86 | 0.26 | (-0.30 ; 0.82) | 0.36 |
| | | G/G vs T/T | 0.08 | (-0.77 ; 0.93) | 0.86 | -0.88 | (-1.89 ; 0.12) | 0.08 | -0.40 | (-1.05 ; 0.24) | 0.22 |
| *TAS2R42* | rs5020531 | T/C vs C/C | 0.36 | (-0.26 ; 0.98) | 0.26 | 0.67 | (-0.05 ; 1.40) | 0.07 | 0.57 | (0.09 ; 1.05) | 0.018 |
| | | T/C vs C/C | 0.31 | (-0.54 ; 1.16) | 0.48 | 0.44 | (-0.74 ; 1.62) | 0.46 | 0.40 | (-0.30 ; 1.09) | 0.27 |

## Supplementary table S5. Genes and SNPs selected in the study

| Chr. | Gene | SNPs | Alleles | Most common annotation |
|---|---|---|---|---|
| **Chr. 1** | | | | |
| | *TAS1R3* | rs35744813 | T/C | nearGene-5 |
| | *CA6* | rs2274333 | G/A | missense |
| | *TAS1R2* | rs4920566 | G/A | intron region |
| | *TAS1R2* | rs35874116 | G/A | missense |
| **Chr. 5** | | | | |
| | *TAS2R1* | rs41467 | T/G | non-coding |
| | *TAS2R1* | rs2234233 | T/C | missense R206W |
| **Chr. 7** | | | | |
| | *GNAT3* | rs7792845 | G/A | closer to gene |
| | *TAS2R16* | rs6466849 | A/G | non-coding |
| | *TAS2R16* | rs860170 | G/A | missense H222R |
| | *TAS2R16* | rs978739 | G/A | non-coding |
| | *TAS2R5* | rs2227264 | T/G | missense S26I |
| | *TAS2R38* | rs10246939 | C/T | missense I296V |
| | *TAS2R38* | rs1726866 | T/C | missense V262A |
| | *TAS2R38* | rs713598 | G/C | missense A49P |
| | *TAS2R39* | rs4726600 | G/A | non-coding |
| | *TAS2R40* | rs534126 | T/C | non-coding |
| | *TAS2R60* | rs4595035 | T/C | synonymous R310R |
| | *TAS2R41* | rs10278721 | T/C | missense P127L |
| **Chr.12** | | | | |
| | *TAS2R7* | *rs2588350* | G/A | non-coding |
| | *TAS2R9* | rs3741845 | T/C | missense V187A |
| | *TAS2R14* | rs11610105 | G/A | non-coding |
| | *TAS2R50* | rs1376251 | T/C | missense C203Y |
| | *TAS2R50* | rs7136962 | T/C | nearGene-5 |
| | *TAS2R20* | rs10772408 | G/A | non-coding |
| | *TAS2R19* | rs12313469 | G/A | cds-synon |
| | *TAS2R31* | rs10845296 | T/C | non-coding |
| | *TAS2R46* | rs2708381 | G/A | nonsense |
| | *TAS2R30* | rs2599404 | T/G | missense F252L |
| | *TAS2R42* | rs5020531 | T/C | missense F196S |
